# Dose effect analysis of sodium zirconium cyclosilicate in hemodialysis patients

**DOI:** 10.1111/hdi.12983

**Published:** 2021-12-19

**Authors:** Bruce Spinowitz, Kieran McCafferty, Steven Fishbane, Masafumi Fukagawa, Nicolas Guzman, Martin Ford, Anjay Rastogi, Sunil Bhandari

**Affiliations:** ^1^ Department of Medicine NewYork‐Presbyterian Queens Queens NY USA; ^2^ Department of Nephrology Barts Health NHS Trust London UK; ^3^ Department of Medicine Donald and Barbara Zucker School of Medicine at Hofstra/Northwell Great Neck NY USA; ^4^ Division of Nephrology, Endocrinology and Metabolism, Department of Internal Medicine Tokai University School of Medicine Isehara Japan; ^5^ Global Medicines Development AstraZeneca BioPharmaceuticals Research and Development Gaithersburg MD USA; ^6^ Department of Renal Medicine King's College Hospital NHS Trust London UK; ^7^ Honorary Senior Lecturer, Faculty of Life Sciences and Medicine Kings College London UK; ^8^ UCLA CORE Kidney Program University of California Los Angeles Los Angeles CA USA; ^9^ Department of Renal and Transplant Medicine Hull University Teaching Hospitals NHS Trust Hull UK

**Keywords:** dose, fluid balance, hemodialysis, hyperkalemia, potassium, sodium zirconium cyclosilicate

To the Editor:

DIALIZE was an international, randomized, double‐blind, placebo‐controlled, Phase 3b study that evaluated sodium zirconium cyclosilicate (SZC), a highly‐selective non‐absorbed potassium binder, in patients with end‐stage kidney disease (ESKD) who had persistent hyperkalemia despite maintenance hemodialysis.^1^ In DIALIZE, SZC reduced pre‐dialysis serum potassium after the long interdialytic interval (LIDI) compared with placebo and was well tolerated.^1^ Recent recommendations suggest that data describing the efficacy and safety of potassium binders in this patient population should be expanded.^2^

Patients with ESKD tend to accumulate fluid, and achieving fluid balance is a vital outcome of hemodialysis.^3^, ^4^ High interdialytic weight gain (IDWG) and ultrafiltration rate (UFR) during hemodialysis are associated with adverse cardiac outcomes, morbidity, and mortality.^3^, ^4^ Minimizing these parameters, while optimally controlling systolic blood pressure (SBP) and diastolic blood pressure (DBP), are key goals in hemodialysis management. The zirconium cyclosilicate ring of SZC exchanges bound hydrogen and sodium ions for potassium and ammonium in a 1:1 ratio.^5^ A potential clinical concern of SZC treatment is how much sodium is released during potassium exchange and its impact on fluid retention.^6^ Each 5 g dose of SZC contains 400 mg of sodium;^7^, ^8^ thus, the maximum sodium amount in each dose is 17.4 mmol, and it is unlikely that all sodium in SZC is exchanged.^6^ In DIALIZE, no consistent, clinically relevant effects on fluid balance variables, including IDWG and blood pressure, were observed with SZC overall.^7^, ^8^ This *post hoc* analysis aimed to assess the impact of increasing doses of SZC on fluid balance parameters in the DIALIZE study.

Full details of the DIALIZE study have been published elsewhere.^1^ The study was performed in accordance with the Declaration of Helsinki, the International Council for Harmonisation, and Good Clinical Practice, and all participants provided written informed consent. The study comprised an 8‐week treatment period, during which patients underwent 4 weeks of dose titration, followed by 4 weeks of evaluation on a stable dose. After randomization (1:1) to receive an oral starting dose of 5 g SZC or placebo once‐daily on non‐dialysis days (4 days/week), the doses of SZC and placebo were adjusted weekly in 5 g increments (up to a maximum dose of 15 g once‐daily) over 4 weeks to attain pre‐dialysis serum potassium concentration of 4.0–5.0 mmol/L after the LIDI. This *post hoc* analysis of the safety analysis population (N = 195) assessed mean changes from baseline (visit 1, day −7) to end of treatment (EOT) in IDWG (kg; measured after the LIDI), UFR (mL/kg/h), SBP (mmHg), and DBP (mmHg). EOT was visit 15 (day 57) for IDWG and UFR, and visit 14 (day 50) for SBP and DBP. Analyses were stratified according to the SZC dose administered at the last visit of the dose‐titration period (visit 11, day 29), as dose adjustment to achieve target serum potassium concentration during the dose‐titration period was a confounding factor. Findings in the placebo group overall are also shown.

During the 4‐week evaluation period, 96 patients received SZC at stable doses of 5 g (n = 38), 10 g (n = 41), and 15 g (n = 17), and 99 patients received placebo. Baseline (visit 1, day –7) characteristics were generally balanced between the groups, except for: mean age was higher in the placebo group (60.4 years) versus the SZC 5 g, 10 g, and 15 g groups (54.5–57.5 years); there were fewer female patients in the SZC 15 g group (29.4%) versus the SZC 5 g (42.1%), 10 g (46.3%), and placebo (41.4%) groups; and mean bodyweight was higher in the SZC 15 g group (83.8 kg) versus the SZC 5 g (73.4 kg), 10 g (73.0 kg), and placebo groups (70.0 kg).

Fluid balance parameters at baseline are shown in Table 1. Mean IDWG, SBP, and DBP at baseline were comparable between the SZC 5 g, 10 g, and placebo groups (Table 1), and were higher in the SZC 15 g group (3.6 kg, 156.3 mmHg, and 89.7 mmHg, respectively). Mean UFR at baseline was higher in the SZC 10 g group (10.620 mL/kg/h) compared with the other treatment groups (Table 1). Mean changes in fluid balance parameters from baseline to EOT are shown in Table 1. Mean changes from baseline in IDWG and UFR were not clinically meaningful between the SZC 5 g (0.0 kg and −0.770 mL/kg/h), 10 g (0.4 kg and –0.030 mL/kg/h), 15 g (−0.1 kg and 0.306 mL/kg/h), and placebo groups (−0.1 kg and 0.396 mL/kg/h). Mean SBP and DBP decreased from baseline to a greater extent as SZC dose increased from 5 g (−1.6 and −0.2 mmHg) to 10 g (−1.8 and −3.2 mmHg) and 15 g (−9.4 and −7.8 mmHg); mean changes from baseline with placebo were 1.3 and −0.1 mmHg, respectively (Table 1, Figure 1).

**TABLE 1:** Baseline values and changes from baseline to EOT in fluid balance parameters

Parameter, mean (SD)	SZC 5 g (n = 38)	SZC 10 g (n = 41)	SZC 15 g (n = 17)	Placebo (N = 99)
IDWG, kg				
Baseline^a^	2.9 (1.4)	2.9 (0.9)	3.6 (1.7)	2.9 (1.6)
Change to EOT^b^	0.0 (1.4)	0.4 (1.2)	−0.1 (1.3)	−0.1 (1.6)
UFR,^c^ mL/kg/h				
Baseline^a^	9.641 (3.980)	10.620 (3.733)	9.601 (3.335)	9.122 (4.387)
Change to EOT^b^	−0.770 (3.378)	−0.030 (2.660)	0.306 (2.844)	0.396 (3.267)
SBP, mmHg				
Baseline^a^	147.3 (27.2)	147.0 (24.8)	156.3 (21.3)	144.5 (22.6)
Change to EOT^d^	−1.6 (23.9)	−1.8 (14.5)	−9.4 (24.1)	1.3 (20.5)
DBP, mmHg				
Baseline^a^	79.6 (14.5)	78.5 (14.0)	89.7 (12.4)	78.2 (14.0)
Change to EOT^d^	−0.2 (10.9)	−3.2 (8.6)	−7.8 (14.6)	−0.1 (12.1)

Abbreviations: DBP, diastolic blood pressure; EOT, end of treatment; IDWG, interdialytic weight gain; SBP, systolic blood pressure; SD, standard deviation; SZC, sodium zirconium cyclosilicate; UFR, ultrafiltration rate.

^a^ Visit 1, day −7.

^b^ Visit 15, day 57.

^c^ Calculated as: actual ultrafiltration (mL)/pre‐dialysis weight (kg)/dialysis duration (h).

^d^ Visit 14, day 50.

**FIGURE 1:**
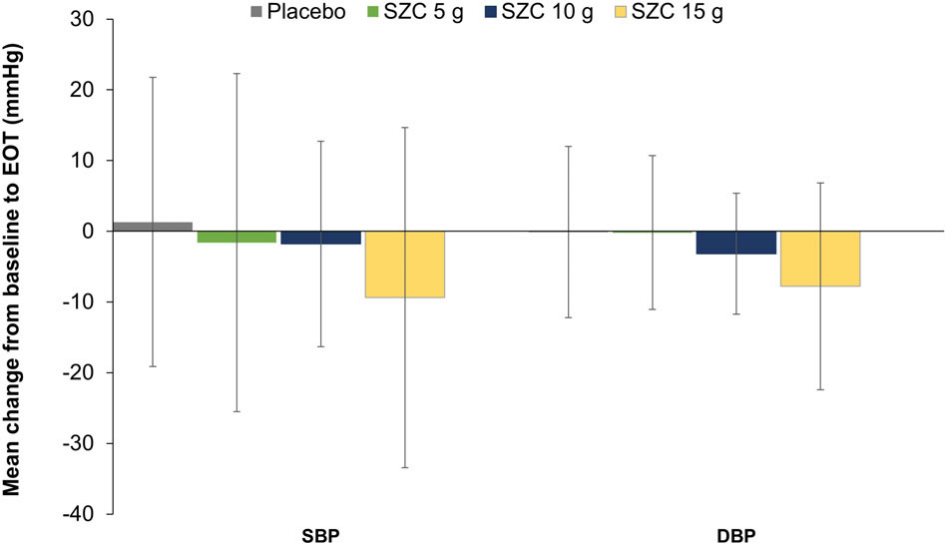
Changes from baseline to EOT (visit 14, day 50) in blood pressure parameters. DBP, diastolic blood pressure; EOT, end of treatment; SBP, systolic blood pressure; SZC, sodium zirconium cyclosilicate [Color figure can be viewed at wileyonlinelibrary.com]

Overall, no consistent, clinically meaningful, dose‐dependent increases from baseline in IDWG or UFR were observed with SZC. The greatest reductions from baseline in SBP and DBP were observed with SZC 15 g. This observation is likely due to these patients having higher baseline SBP and DBP values, in addition to a smaller sample size than for the other dosing groups, and so these reductions in blood pressure likely indicate regression to the mean which is unrelated to SZC. The impact of SZC dose on serum bicarbonate was not assessed here; however, increases in serum bicarbonate levels have been shown with SZC in a dose‐dependent manner in Phase 3 studies of patients with hyperkalemia.^9^ Indeed, in patients on maintenance dialysis in DIALIZE, similar increases from baseline in serum bicarbonate were observed with SZC overall at Day 57 (mean [SD]: SZC +0.5 [2.3] mmol/L, placebo −0.3 [3.1] mmol/L).^1^ This observed increase in serum bicarbonate is likely due to SZC binding of gastrointestinal ammonium. The impact of dose titration in each SZC dose group and the *post hoc* nature of the analysis should be considered when interpreting these findings. In conclusion, these findings suggest that SZC dose can be adjusted from 5 g to 15 g on non‐dialysis days to attain pre‐dialysis serum potassium concentrations of 4.0–5.0 mmol/L without affecting safety with respect to short‐term fluid balance.

## CONFLICTS OF INTEREST

Bruce Spinowitz received research grants, lecture fees, and/or consulting fees from AstraZeneca, Akebia, Reata Pharmaceuticals, and Fresenius Medical Care. Kieran McCafferty is an academic grant holder and an advisory board member for AstraZeneca. Steven Fishbane received research support and consulting fees from AstraZeneca. Masafumi Fukagawa received consulting fees and lectures fees from AstraZeneca Japan. Nicolas Guzman is an employee of AstraZeneca. Martin Ford received travel support from Amgen and AstraZeneca and is an advisory board member for AstraZeneca. Anjay Rastogi received research or travel support from and/or is a speaker, consultant, or advisory board member for AstraZeneca, Relypsa, Fresenius Medical Care, Sanofi, Kadmon, AMAG, Otsuka, Genzyme, GSK, Omerus, Janssen, Reata Pharmaceuticals, Ironwood, and Amgen. Sunil Bhandari has given lectures and participated in an advisory board for AstraZeneca, has given lectures sponsored by Vifor Pharma, and has received travel support from AstraZeneca and Vifor Pharma.

## AUTHOR CONTRIBUTIONS

All authors contributed to the data interpretation, critically reviewed the manuscript, approved the final version, and accept accountability for the overall work.

## Data Availability

Data underlying the findings described in this manuscript may be obtained in accordance with AstraZeneca's data sharing policy described at https://astrazenecagroup-dt.pharmacm.com/DT/Home.
